# Health Literacy as Communicative Action—A Qualitative Study among Persons at Risk in the Context of Predictive and Preventive Medicine

**DOI:** 10.3390/ijerph17051718

**Published:** 2020-03-05

**Authors:** Laura Harzheim, Mariya Lorke, Christiane Woopen, Saskia Jünger

**Affiliations:** 1 Cologne Center for Ethics, Rights, Economics, and Social Sciences of Health (CERES), University of Cologne and University Hospital of Cologne, Universitätsstraße 91, 50931 Cologne, Germany; christiane.woopen@uni-koeln.de (C.W.); saskia.juenger@uni-koeln.de (S.J.); 2 Research Unit Ethics, Institute for the History of Medicine and Medical Ethics, Faculty of Medicine, University of Cologne and University Hospital of Cologne, Universitätsstraße 91, 50931 Cologne, Germany

**Keywords:** health literacy, persons at risk, ethnographic approach, health sciences, qualitative research, perceptions of health and disease, critical health literacy, shared decision making, communicative action

## Abstract

Predictive and preventive medicine play an increasingly important role in public debates on health, providing cutting-edge technologies with the potential to measure and predict individual risks of getting ill. This leads to an ever-expanding definitional space between being “healthy” and being “ill”, challenging the individual’s everyday life, attitudes and perceptions towards the self and the process of health-related decision-making. “How do the condition of ‘being at risk’ and individual health literacy interrelate?” is the leading question of the current contribution. Drawing on empirical qualitative data, collected by means of narrative interviews with persons at risk in four clinical fields, a bottom-up ethnographic and health sciences perspective on health literacy (with an emphasis on critical health literacy) is employed. The findings will be embedded within theoretical approaches dealing with power relations and communication in healthcare encounters, particularly Habermas’ theory of communicative action. The core outcome of our study is a concept for an overarching model of health literacy in the context of health-related risk prediction across indications, based on empirical insights gained through interpretative analysis of the four clinical domains.

## 1. Introduction

Being confronted with a health risk entails the solicitation to deal with risk-related information. When conducting research on the phenomenon of being at risk, it is therefore important to consider the evolving possibilities of predictive and preventive medicine, and their effects on individual needs with respect to information and support in decision-making processes. Communication between health care professionals and persons at risk is an essential element in this course. In the following, we wish to briefly introduce the meaning of the notion of ‘being at risk’, the role of health literacy, and the relevance of interpersonal communication in the setting of predictive medicine.

### 1.1. Being at Risk

Technical innovations in the field of predictive and preventive medicine allow for early detection of individual risk factors concerning a constantly increasing number of diseases. This presents health professionals, patients and their relatives with new manifold challenges. From a patient’s perspective, to be confronted with a (suspected) increased risk of developing a certain illness does not only mean to correctly understand and appraise the medical ‘objective’ risk prognosis, but also to manage the emotional confrontation with the new, identity-relevant role of being a “person at risk”. While the predicted event lies in the future and may not cause any current strain or suffering, individuals need to make choices and/or take action in the present, with immediate effect and sometimes serious intrusion upon their everyday life and quality of life. This condition and its medical, psychological and social consequences place special demands on individual health literacy (HL). Risk and health information need to be managed by the individual, transferred into the process of (shared) decision-making (SDM) in order to interact effectively with physicians, and integrated into one’s subjective everyday life. The focus of this contribution is therefore at the interface between health risk, health literacy, and communication.

### 1.2. Health Literacy

Today’s health-society [1] promotes an ideal image of self-effective, proactive patients who are able to make informed decisions successfully managing their own health and/or disease. The concept of health literacy (HL) has become a benchmark for doing health research for people and with people in the aspiration of a healthier society. According to the integrated definition developed by the European Health Literacy Consortium [2,3], “Health literacy is linked to literacy and entails people’s knowledge, motivation and competences to access, understand, appraise and apply health information in order to make judgements and take decisions in everyday life concerning health care, disease prevention and health promotion to maintain or improve quality of life during the life course” [2] (p. 3), [4] (p. 4). Based on this definition, risk related HL can be considered as the ability to access and understand information on risk factors for health, derive their meaning, interpret and evaluate this information and to make informed decisions with regards to risk factors for health [2].

Despite the ubiquitous presence of HL, there is a fundamental lack of consensus about the definition, the conceptualisation, and the scope of the term [5]. For the purpose of this contribution, we are drawing on a more comprehensive, resource-oriented approach [6]; following Samerski, we conceive of HL as a situational, multidimensional, and dynamic process, including a variety of sources and forms of knowledge, which is co-produced in social relations [7]. In this light, HL can be considered as being closely interwoven with processes of shared decision making (SDM) concerning medical interventions. For example, Smith et al. [8] found that people with higher health competence perceived decision-making as a joint negotiation process, which they could actively shape, while persons with lower levels of HL appeared to engage in the decision-making process less autonomously; rather than actively participating, they were more likely to accept the doctor’s recommendation. At the same time, subjective HL and the involvement in SDM process have a positive impact on the satisfaction with medical care, the compliance and the success of medical interventions [9]. HL therefore plays an essential role in the extent to which people are involved in decisions about medical interventions and thus how satisfied they are with medical treatment. There is hence a widespread demand of promoting HL in terms of involving patients more actively in decision-making in the context of medical consultations [8,9,10,11,12].

### 1.3. The Value of Communication

As much as patients are facing challenges of navigating through complex information upon anticipated health conditions, risks and chances of predictive diagnosis and disease prevention, healthcare professionals are expected to provide them with full and comprehensive information about individual disease risks and preventive options, ensuring an environment where there is enough time, communication and empathy to mutually find individual-sensitive solutions. There is a shift in medical consultation models from the traditional, paternalistic patient-physician-relationship, where doctors make recommendations and patients give their consent, towards the model of SDM, meaning the exchange of information and preferences about diagnostic and therapeutic procedures between patient and physician [13]. There is also an ethical claim towards healthcare professionals with respect to risk-adjusted patient-information and preventive decisions: While every medical intervention is per se an act of bodily harm and only legal when informed consent is given by the patient, interventions in (still) healthy persons for the sake of pre-clinical measures and preventive treatment require special accuracy and comprehensibility of information provided by the physician [14]. Ishikawa & Kiuchi [15] almost ten years ago noted with respect to the role of HL in health communication that the concept of HL should be examined not only as an individual set of skills but also “in terms of the interactional processes between individuals and their health and social environment“ [15] (p. 1). We believe that by now this approach remained highly underrepresented in the research on HL and are convinced that it may be crucial not only to better understand the interrelation between individual and organisational HL [16] (especially in context of risk), but also for the development of tools, instruments and interventions which can lead to an improved HL on an individual and a social level. In consequence, when striving for a bottom-up approach^1^ to HL in “persons at risk”, attention needs to be directed to the communicative character of the concept, situating it within the theoretical framework of SDM and health communication. Our approach is informed by a perspective on the interactional dynamics and the power relations that are shaping a communication process and its outcomes. In particular, Habermas’ theory of communicative action is considered as fruitful for understanding how HL is co-constructed in healthcare settings, and as a framework for encouraging critical health literacy. This theory proposes claims of validity for judicious communication and mutual understanding, based on equal opportunities concerning the initiation of and participation in dialogue, and contributing to arguments and interpretations. Furthermore, it allows for insight into the individual’s lifeworld without neglecting the organisational context, emphasising the interrelation between both. This concept, including its link to (critical) HL, will be elucidated in more detail in the discussion of our findings.

### 1.4. Aims and Research Questions

The overarching goal of this contribution is to approach HL in its interactional dimension. Employing a health sciences and an ethnographic research perspective, we aim at providing a new bottom-up definitional approach to the concept of HL in the context of health risk, with an emphasis on critical HL. The leading questions of the research project are: (1) How is a person’s HL interrelated with the condition of ‘being at risk’? (2) What kind of HL do people need in order to manage their health risks (from a bottom-up perspective)? (3) How can HL be promoted in order to support individuals in the process of SDM and of transferring medical risk information into their lifeworld? The aim is not only to enrich the body of research on the theoretical and conceptual underpinnings of HL [17,18,19] and to contribute to the understanding of HL in the context of risk, but also to provide an empirical foundation for the development of interventions for communication about risk in healthcare settings [15,20], hereby improving HL both on an individual and on an organisational level.

## 2. Materials and Methods

This qualitative study is part of the project Health Literacy of Persons at Risk – From Information to Action (RisKomp) which investigated the role of HL in persons with an increased risk of developing a disease in one of four exemplary clinical fields (Alzheimer’s dementia (AD), familial breast and ovarian cancer (FBOC), coronary heart disease (CHD) and psychosis (PSY)). The choice of these exemplary clinical fields was based on the fact that they allowed a focus on disease patterns with epidemiological relevance in oncology, neurology, cardiology and psychiatry, and thus the exploration of risk perception and HL relevant factors in the field of mental as well as physical disease. By considering diseases with a diverse definition of risk factors and different methods of creating risk profiles (including symptom assessment as well as biomarker and genetic testing), it was possible to include diverse risk patterns in the analyses. A further gain of knowledge was made possible by the spectrum of prevention opportunities and therapeutic approaches, which can either prevent the onset or have a positive effect on the course of a disease. The intervention options concerning the four indication areas range from surgical or medicinal, psychotherapeutic and educational, to no effective medical prevention option so far in regard to Alzheimer’s dementia. This allows taking into account strategical deliberations of persons at risk; depending on health-related future scenarios they were confronted with in the course of predictive procedures. The systematic reviews conducted in the first phase of the project provide an overview of the research landscape and current empirical evidence concerning the role of HL with respect to an increased risk in each of the four clinical fields; in addition, they revealed open questions and directions for future research [21,22,23]^2^.

The study was planned and conducted in close collaboration with partners in the cooperating specialist centres at the University Hospital Cologne (Appendix A)^3^. Ethics approval was obtained in March 2018 (registration number 18-014) by the Medical Faculty of the University of Cologne.^4^

### 2.1. Sampling and Recruiting Procedure

For the recruitment of interview candidates, indication specific inclusion and exclusion criteria (Appendix B) were defined in cooperation with the specialist centres for genetic testing or preclinical diagnosis of the University Hospital of Cologne. In a first step, for each clinical field, risk profiles were determined based on current medical evidence (e.g., a particular type of genetic risk or a combination of genetic, physiological, and behavioural risk factors). The aim of this purposive sampling strategy was to enclose a maximum variety of risk manifestations for each clinical field [24]. According to the in- and exclusion criteria, the clinical staff started recruiting the individual participants based on convenience sampling. There is no consensus about the ideal sample size [25]; while it is often necessary to specify a certain number of interviews for ethics approval and funding calculation, the inclusion of ten participants per clinical field was envisaged (40 in total). This number was based on the project aim, the research question, the chosen study design, as well as the available personal and institutional resources.

The collaborating clinics supported us in recruiting participants by pre-screening their patients’ profiles with regard to the in- and exclusion criteria, by handing out brief information about the research project to the potential participants, by imparting their contacts to the project team, and by providing facilities for the interviews. In the field of coronary heart disease, in addition to cooperating with teaching practices of the University Hospital Cologne, online recruitment strategies, social media, public displays as well as the contacting of support-groups and relevant organisations were used as recruitment strategies. The clinical staff arranged the first contact with potential participants; all further steps like providing detailed study information, arranging the interview appointment, conducting the interview, and any further communication with the participants was at the authors’ responsibility.

### 2.2. Data Collection

To answer the research questions, we chose a qualitative research design which incorporates three pools of data: (1) narrative interviews on risk and health, (2) body sketches visualising embodied perceptions of risk and illness, and (3) ethnographic data based on notes and memos concerning reflexivity and the research relationship, created before, during and after the interview.

#### 2.2.1. Narrative Interviews

The narrative interview, chosen to be a main source of data collection in this research project, is a methodology of qualitative social research to gain insights into the interviewees’ personal experiences, feelings and subjective relevancies in a context of interest. The idea of the narrative interview is to let the interviewees tell their “stories” and herewith communicate their perspective on a subject without narrowing the course of information by giving a direction of conversation with a standardised interview guide [26]. Narrative interviews were deemed suitable for this project since they allow for a bottom-up approach to concepts such as risk and HL. Letting these be defined by the persons’ individual perceptions, appraisals, and preferences, narratives can inform the development of theory which is grounded in empirical data. Being interested in HL relevant factors from the perspective of persons at risk, narrative interviews using a flexible topic-guide were the instrument of choice. This approach follows the principle of narrative interviews, starting the conversation with an open introductory question, but sharpening its focus by context-specific in-depth questions (incorporated in the topic guide), pre-defined by the researcher alongside the subject of interest [27]. The interviews started with a question about the first confrontation with being at risk of developing a certain disease^5^ [28]. With reference to the interviewees’ narratives, in-depth questions were asked on access, understanding, appraisal and application of risk-related health information. The interviews were concluded when no new themes or stories were raised, and upon the researcher’s explicit invitation to think of any further potentially relevant issues that the interviewee may wish to describe.

The interviews were audio-recorded with the participants’ given consent. The audio material was transcribed verbatim, and transcripts served the text-based analysis of the interviews using MAXQDA 2018 [29]. In addition, sociodemographic questionnaires were included into data analysis that had been handed out to and filled in by participants before the interviews.

#### 2.2.2. Embodied Perceptions of Risk and Illness

At the end of each interview, participants were asked to perform a body-mapping exercise. A sheet of paper showing an empty body sketch was given to participants, who were subsequently asked to depict their feelings about being at risk of developing a disease. Interviewees had the opportunity to comment on their drawings if they felt the needed to do so. The method of body-mapping is an approach with which insights in the individual’s embodied realities can be gained, and is used in health sciences [30,31]. Body-mapping allows the non-verbal and creative expression of perceptions, personal feelings and experiences and is therefore suitable for the inductive, bottom-up approach aimed at in this study.^6^

#### 2.2.3. Ethnographic Data Concerning the Research-Relationship

During the interviews, the researchers took field notes concerning content, non-verbal communication, atmosphere and their own experiences and feelings during the interviews using a self-reflection tool developed for the purpose of this study (notes and memos). The documentation and reflection of one’s own feelings and observations during and after interviews is a common practice to make the researchers’ subjectivity comprehensible and transparent, which is a key quality criterion in qualitative social research [32,33]. The notes and memos were included in the data analysis and methodical reflection of the research process.

### 2.3. Data Analysis

The analysis process of this study is embedded in the overarching approach of the Reflexive Grounded-Theory-Methodology [34], and mirrors an iterative process of three analytical steps: (1) analysis of data (narratives, body-maps and ethnographic data) for each clinical field separately, using a field-specific coding system; (2) interpretative analysis of the findings in all four clinical fields, developing a new integrated coding system, and (3) interdisciplinary data validation and cross-check analysis.

#### 2.3.1. Analysis of the Narratives, Body-Maps and Ethnographic Data for Each Clinical Field Using a Field-specific Coding System

In this first analytical step, data in each clinical field were analysed separately by different team members in an iterative process parallel to conducting subsequent interviews. The goal was to identify categories that are specific to the risk of disease in the respective clinical field, avoiding direct comparisons between data in the process of collection and first analysis. With the exception of CHD, thematic saturation^7^ [35,36] was reached before completing analysis for all envisaged (n = 10) interviews in each clinical field; i.e., the main categories remained stable after having analysed approximately six to seven data sets in the respective clinical field, even when including further interviews in the analysis. During the process of open coding, we created a coding system embracing the different data types (narratives (verbal), body maps (visual), and field notes (reflexive)), which provided insights into different emic interpretations of risk, health and HL. Furthermore, through the integration of field notes into the analysis it was possible to address the question of researchers’ subjectivity and make it as visible as possible in the analytical process. In the process of axial and selective coding, we searched for interconnections with the concept of HL, both describing it from the patients’ perspective and relating it to existing definitions of the concept from the literature. Through this triangulation of methodology and theory [37], we aimed to emphasise the ethnographic, bottom-up character of the research.

#### 2.3.2. Interpretative Analysis of the Findings of All Four Clinical Fields, By Developing a New Integrated Coding System

**Figure 1:**
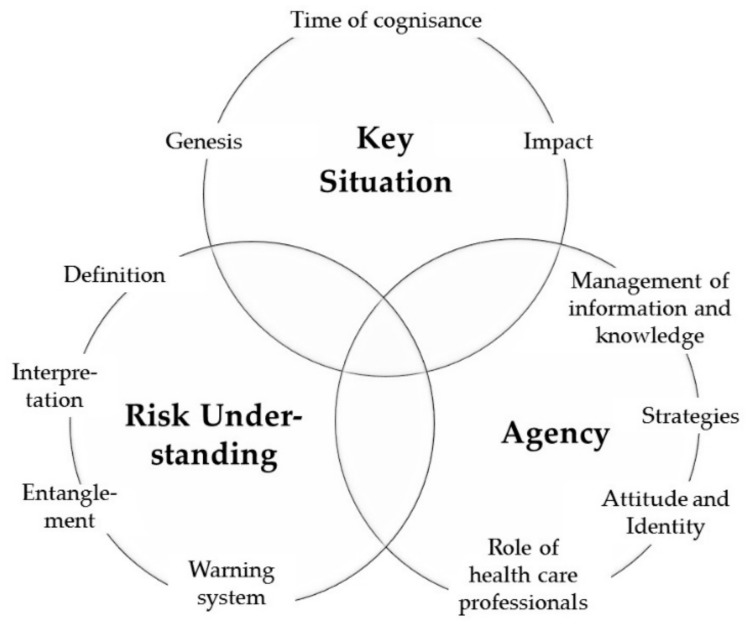
Central categories of dealing with disease risk: key situation, risk understanding, and agency.

In the second stage of analysis, we conducted an interpretative analysis across coding systems, integrating the codes of all four clinical fields. Due to the restricted number of interviews in the field of CHD, interpretation was considered with reservation. Both indication-specific and cross-indication findings regarding HL-relevant factors in persons with an increased risk of disease, were compared and discussed within the research team, taking into consideration different perspectives from our disciplinary backgrounds (ethnology, psychology, and health sciences). In this way, we were able to identify categories and relations relevant for all four fields as well as those themes that are specific for each clinical field. For the purpose of this article, we created an overarching category system (Figure 1, Section 3.2) which emerged during the analysis across the clinical fields based on the research questions stated above, and the previously gained in-depth insights in the different fields. Through this approach, decision making processes of people with an increased disease risk regarding the use of early diagnostic procedures and preventive interventions can be reconstructed. This allows for reflecting on conducive models of risk communication in connection with health behaviour and contributes to the theoretical foundation of the concept of HL.

#### 2.3.3. Interdisciplinary Data Validation and Cross-Check Analysis

In the final analytical phase, we aimed at researcher, methodological and theory triangulation, and interpreted and validated the interpretative analysis performed in the second analytical step. For this purpose, we organised interdisciplinary researcher discussions in order to relate the concepts which arose from the empirical data to existing theoretical frameworks in different academic fields, and to test the applicability of the developed theoretical considerations across the four clinical fields.

## 3. Results

In the following, we will shortly introduce the database of the research project, and present the central findings of our study along the structure of the main categories and sub-categories that we identified during the interpretative analysis.

### 3.1. Database

The interviews were conducted between April 2018 and August 2019. In total, 34 out of the envisaged 40 interviews were completed. During one interview in the AD group, it turned out that at the time of the study, the respondent already had dementia (exclusion criterion). Therefore, 33 interviews were included in the data analysis (a detailed presentation of the participants’ characteristics is provided in Appendix C). In the area of CHD, despite various recruitment strategies during the period of data collection, no more than three persons could be recruited to participate in the research project (Table 1). (The recruiting strategy and possible reasons for the low response rate compared to the other clinical fields will be methodologically reflected in the further course of the evaluations).

Audio material of approximately 34 h, 1036 pages of transcripts, 33 questionnaires and 31 body-maps, plus the researchers’ memo material constituted the data sources used in the data analysis process (Appendix D).

### 3.2. Main Categories

Hereinafter, our findings concerning HL-relevant factors for people at risk of developing a disease, and HL-related aspects in order to manage these risks, will be presented. The results focus on the respondents’ subjective risk and disease theories, as well as on their analytical-reflexive and emotional-intuitive interpretation systems. We identified three central categories which refer to key situations of risk perception, the processing and understanding of disease risk, and risk-related agency of “persons at risk” (Figure 1).

#### 3.2.1. Key Situations

In the context of this study, key situations describe a distinct moment or an extended period of the ideational realisation of one’s risk. This is determined by a person’s biographical background, intuition, and degree of self-reflection. Whether a person is confronted with the risk of developing a disease for the first time or has been confronted with the idea of risk directly or indirectly for years, has an influence on how the risk is dealt with, and on the decision-making processes regarding early diagnostic or preventive measures. In the following, features of key situations identified in the interviewees’ narratives will be described. The identified key situations can be differentiated along the process of being confronted with risk, in terms of the emergence of a risk feeling, the time of cognisance of risk, and the impact of key situations.

##### Genesis

Genesis describes the development that gave the initial impetus for dealing with the concept of risk, and ultimately participating in early diagnostic procedures. Respondents describe an omnipresent risk idea or an intuitive risk perception over a longer period of time to be the reason for the participation in early diagnostic procedures as a form of active engagement with their own health-related future:

“That’s a little strange because, um … that was for me, well breast cancer has always been an issue for me.”

(FBOCP06)

“Yeah, I kind of want to know what that is and why. [...] Just to have the certainty.”

(ADP08)

“And it’s nothing that has uh just been there for three months, it has always been there. Well, it didn’t just appear like three months ago, it has always been there.”

(PSYP10)

##### Time of Cognisance

The time of cognisance means the moment or period of time of risk confrontation in which participants became aware of their risk. According to interviewees’ descriptions, a moment of confrontation in the sense of a sudden awareness of a risk can be caused by biographical upheavals (relocations, life stage changes, crises) or chance findings during routine examinations. Dealing with disease risk over a longer period of time can be the case due to disease experiences in the family or the observation of symptoms that people may associate with the development of diseases and interpret them as a potential precursor (forgetfulness, changes in perception, cardiovascular complaints): “Sometimes I don’t have any problems at all and sometimes I think ‘It can’t be true that you don’t remember this anymore!’.” (ADP07).

Facing an increased disease risk, whether in a moment of confrontation or over a period of becoming aware, entails a transition or a turning point in the lives of the participants of this study, with characteristic consequences for their identity formation, lifestyle or future perspectives. The interviewees’ narratives contain detailed episodic accounts of the situations or periods of becoming aware and inescapably realising that ‘something is wrong’.

“Um, and then, as really the most relevant moment was when I was at the North Cape and the big turquoise wide sea was in front of me, the people around me were all happy, there were some plants around me you can’t find anywhere else, and I just didn’t feel anything.”

(PSYP01)

“I was still young and thin and thought: ‘How could that be, diabetes type two, you get that at seventy or eighty or so and if you get it before then, it’s because you’re a rather chubby person!’ I was really shocked. Because I expected everything, but not that I would become diabetic at the age of fifty or in my early fifties.”

(CHDP01)

##### Impact

Respondents described key situations to have an existential impact on their considerations and planning with relevance for their life course, their identity, and their family planning: “The consequence of this, if I think about my husband and myself, would be family planning.” (FBOCP10), their professional context: “I already told my superior to consider me as a risk factor” (ADP04) or their view on future life in general: “There are days where I only look at the black side of my future.” (ADP07).

The impact of key situations on people’s perceptions or actions can be of a positive and motivational nature: “I want to stay healthy you know? So living healthily is my contribution to not getting high blood pressure.” (CHDP03). At the same time, key situations can have a negative-destructive impact: “All I feel is fear of getting dementia. […] It is in every cell.” (ADP01).

This is of central importance with respect to patient information and risk communication. Consultation in the context of early diagnostic counselling and intervention planning can be decisive in terms of the extent to which people who seek advice are motivated and enabled to make health-promoting decisions and actively shape their health development wherever possible.

Key situations can also provide information on peoples’ preferences, skills, and strategies in researching and selecting risk-related health information. Whether, for example, they have been informing themselves about a possible risk of illness for a longer period of time or whether they do not yet know or have not sought any information on the subject at all, sheds light on the scope and the content of information needed. This provides a starting point for medical consultation.

The identification of key situations can also mean orientation for individuals’ lived realities, their biographical experiences, and their socio-cultural embedding of the risk understanding. These background circumstances are pivotal in terms of people’s needs concerning health-related information and decisions.

The available data offer an opportunity to define types of key situations. A typology of key situations with respect to their emergence or time of cognisance (e.g., sudden or foreseeable) and their impact (e.g., shock or confirmation), can serve as an orientation framework in the early diagnostics of risk and preventive praxis, helping professionals to provide risk information based on individual needs.

#### 3.2.2. Risk Understanding

The understanding of risk comprises aspects of subjective risk definition and interpretation, as well as the individual’s relative meaning of a disease risk. The following issues were identified with respect to the understanding of risk.

##### Definition – Describing Risk

People have individual definitional concepts of a disease risk. ‘Definition’ in this context refers to metaphors and descriptions participants use to name their risk. People, for instance, describe their risk as a “time bomb” (ADP09), a “tattoo” (PSYP10) or a “bookmark” (PSYP10) and thereby reveal risk to be experienced as something threatening, stigmatising or permanent. Defining one’s personal risk means naming it on the one hand; on the other hand, naming it by using metaphorical terms also means applying interpretational concepts to it. The definition and interpretation of risk are therefore closely interwoven and determined by personal disease conceptions. Threat rhetoric used by interviewees with regard to their disease expectations show that experience-based disease images, which for example are associated with decay, hopelessness, strain on relatives or the loss of the social role and one’s own identity, pre-set a definitional framework: “I would like to see my daughter grow up and be an adequate companion for her and not a […] senile one.” (ADP03). The metaphorical description of risk perception and disease conceptions emerging in the context of the body maps, both in visual and in verbal form, provide insightful information about risk-related perceptions or visions of one’s own state of health. Definitional concepts and interpretations of disease risk are crucial in the process of meaning-making [38] concerning potential future health scenarios.

##### Interpretation – Appraising Risk

According to the analysis of the interviews in our study, the appraisal of risk, as illustrated by the following exemplary quotations, is largely determined by personal conceptions of a disease, which in turn are influenced by self-inflicted or externally-intrigued experiences of illness. People who have already experienced the course of a disease, for example by caring for a relative, project these experiences onto themselves and define their own future state of health accordingly: “I know lots of people with dementia in my environment. […] Seeing my friends’ parents. That’s really bad you know.” (ADP05) or “I don’t want not to be pretty anymore. […] I have seen my cousin dying of cancer, she looked so ugly. That was really bad.” (FBOCP05).

Risk knowledge in the present about an anticipated state of health in the future can influence the perceived quality of life. Thus, the boundaries between being healthy and being ill already are blurred by the imaginary confrontation with a disease risk before the actual occurrence or onset of a possible illness. Perceived symptoms, for instance, can entail a disease experience even before the actual manifestation of a condition: “I have all the symptoms!” (ADP01). This influences everyday life and lifestyle: “This fear that they might say ‘Ok, there is something.’ keeps me getting these panic attacks.” (FBOCP02) or “I definitely try my best to live more relaxed, and not to let it get any worse.” (PSYP05).

“Healthy” and “sick” are thus redefined and persons at risk are confronted with identity-relevant changes. HL of persons with an increased risk of illness - their resources and motives to deal with and apply risk-related health information - depends on their ability to integrate the risk status into their own reality and to accept or actively reject it as part of their identity.

In the individuals’ perceptions, the perceived risk prevails over the actual (statistical) probability score: “And in the end we are people, not statistics.” (FBOCP09). For example, the results of early diagnostic examinations may contradict the feelings of those seeking advice: “I was always, um, totally irritated because of these test results, I have to admit. Because they didn’t reflect at all what I am feeling for myself.” (ADP05). The consequence can be that an existing risk is not perceived as such: „Well, I don’t know. I have been told that I am at risk, so to say. But yeah. That’s all.“ (PSYP02). In this case, people face the conflict of making decisions about a situation of which integration into their lifeworld does not correspond to their own perceptions.

Percentages given to respondents were interpreted subjectively. Risks can be perceived as an omnipresent threat even with a low numerical probability of illness. While early risk detection procedures for some persons can imply the positive effect of an “early warning system” (which will be described in more detail in the following), for others these do not convey a sense of security if they pervasively continue to feel that they are facing a health threat despite the “all-clear signal”. For example, respondents stated that they did not feel any sense of security, even though the result of their predictive examinations did not reveal an increased risk of developing a disease: “I mean, it was a fact that there was something a way it was not supposed to be.” (ADP05). So, the emotional evaluation outweighs the logical interpretation of percentages and factual findings.

##### Entanglements

In addition to such emotional-intuitive interpretation systems, the analytical-reflective approach is of relevance when dealing with the probability of illness (consistent with the model by Slovic et al. [39] on the emotional and analytical handling of risk). The respondents’ statements on the subjectively perceived relativity of risk refer to risk entanglements as well as to vague versus concrete risk conceptions.

For example, the disease risk that respondents were undergoing early diagnostic procedures for, was repeatedly named in connection with parallel existing diseases or disease risks: “Being overweight is part of my biography. I am overweight now […] and I have been overweight as a kid or as a teenager.” (CHDP03). Risks are therefore not perceived and processed as isolated entities but understood as interdependencies with other risks: “It could be that my depression has affected my cognitive condition, couldn’t it?” (ADP04) or “Sure there is a risk when you smoke and drink alcohol.” (FBOCP01).

When dealing with a certain disease risk, persons reflect their own (prospective) health as the totality of various (risk) factors. Reflection in the sense of critical HL [40] goes beyond the differentiated examination of health-related risk information and includes an examination of one’s own lifeworld in terms of values, preferences, habitualisation, and social circumstances. As a practical implication for early diagnostic consultations and treatment, these findings can inform starting points for health-promoting or preventive measures.

Vague versus concrete risk conceptions determine to what extent people adopt a “diagnosed” risk probability and include it in their consideration processes and actions. The perceived degree of abstraction of both the risk and the respective image of disease plays a role here. Our data allow the assumption that risk developments and disease progressions with psychological or mental effects are likely to be experienced as more difficult to “grasp” than risk prediction of diseases with physical consequences, the development of which can be – according to respondents’ appraisal – specified more precisely by means of biomedical parameters: “Subjective cognitive disorder, my God. You have your aches and pains and that’s just one of them.” (ADP03) or “I’m not saying that everybody has mental issues but in some way […] other people do struggle with their everyday life, too.” (PSYP01).

##### Warning System

Irrespective of the degree of abstraction of the risk, people who participate in early diagnostic procedures actively deal with their risk. In this context, risk prediction is interpreted as a kind of early warning system and as a resource in the informed handling of one’s own health. Respondents describe that the medical prediction of their likelihood of disease gives them a feeling of clarity and therefore the opportunity for active prevention – in terms of medical interventions, organisational preparations or lifestyle changes. The last quotation also underscores the blurring between ‘risk’ and ‘disease’ in the interviewees’ accounts.

“That’s why I’m glad I was able to deal with my problems now. [...] And I’m just glad that this bang fortunately caused the discernment that I have to let people help me.”

(PSYP07)

“That was just another piece of the puzzle for me. It was absolutely out of question. Either you want to know or you don’t. I already said before I knew for certain: Just take my breast off!”

(FBOCP04)

“We have decided to downsize a little with respect to our living. Age-appropriate. That as well has to do with my dementia.”

(ADP06)

Our interviewees’ risk narratives reveal the process of understanding, evaluating and applying risk information. Subjective interpretation patterns and relevance systems are crucial for the interpretation of risk and the resulting motivation to act. This negotiation process can be revealed through the narratives of those seeking advice to meet their needs with regard to medical consultation and treatment.

#### 3.2.3. Agency

The category “Agency” refers to individual autonomy and manageability in view of an increased risk of illness. It comprises emotional, cognitive, and behavioural strategies that our interviewees reported in order to (re)construct their capacity to define their situation, to make choices, and to act independently. This includes aspects of information and knowledge management, the role of attitude and identity in dealing with a health-related risk, individual strategies of action, and the role of health care professionals in risk perception and processing.

##### Dealing with Information and Knowledge

Respondents name various sources of information they use to get informed about their disease risk, including articles, studies, TV and books. They also mention the Internet and the social environment to be central media for the exchange of experiences or personal opinions and the search for risk- or disease-related information: “And when you read something like this, what do you do nowadays? Google.“ (ADP03) or „I talk to my husband, he is of great help to me.” (FBOCP03). The finding and understanding of health information does not refer to an isolated source of information but to a construct of several sources of information, which people individually choose and evaluate. The information medium therefore goes beyond the medical setting and the doctor-patient communication setting in early diagnostic procedures. According to the respondents, the primary information strategy is to obtain health information by oneself: “I can only recommend to get as much information as possible.” (ADP04). They critically decide where to look for information: “You have to be careful about where you find your information, right?” (FBOCP01), what information they choose for themselves: “I don’t trust my doctors exclusively anymore.” (FBOCP01), and which information they want to or do not want to deal with: „Sometimes you just don’t want to know it in cold print, you know?” (ADP07). Information seeking and evaluation strategies depend on the individual’s systems of experience and relevance.

##### Attitude and Identity

In terms of attitude and identity, personal competences such as interest, motivation, self-reflection and self-efficacy are essential prerequisites for the way people deal with information about and the personal exposition to health risk. Participation in predictive procedures, for example, is described as self-initiative based on self-observation and self-reflection. With the decision for or against information, examinations, study participation or reporting of findings, a competence for one’s own needs becomes visible, which has to be included and taken into account during counselling.

Of equal importance for attitude and identity in dealing with a disease risk is the social environment. Persons within the social network – family, partners, and friends – directly or indirectly influence decision-making processes with regard to early diagnostic procedures as well as the negotiation processes regarding therapeutic measures. Direct influence exists, for example, when relatives actively encourage participation in diagnostic testing: “Well I have to say, my oldest daughter was the one who told me to see a doctor.” (ADP07). Indirectly, a feeling of responsibility towards relatives may for instance be decisive for a person’s step towards medical risk prediction: “I have three children. I think about them, I don’t really think about me.” (FBOCP02).

While identity and attitudes determine how health risks are dealt with, they can also be influenced by the way risk is conceptualised.

“I am a risk factor.”

(ADP05)

“And then you go like: ‘Oh shit – this is like a tattoo!’ That’s gonna stay for now.”

(PSYP10)

For the promotion of health-literate action in dealing with disease risk, this finding shows that factors such as intrinsic and extrinsic motivation as well as input from the social environment deserve to be taken into account in medical consultations.

##### Strategies

In dealing with a disease risk, our interviewees reported having developed personal strategies. These include, for example, subjective explanatory models and measures to maintain or improve the subjective quality of life. As elaborated further below, explanatory models are both a strategy to process the origin and development of risk within people’s own logical system. At the same time, these models are the starting point for the development of strategies for dealing with a disease risk, which involves the acceptance of a “new” reality and the assumption of the risk status. Health-oriented decisions and lifestyle adjustments can be the result.

With reference to our interviewees, explanatory models of risk were individual and biographical. When negotiating one’s own risk, not only information from outside is taken into account, but also theories of justification which are constructed by the individuals themselves. In this way, they explain their own risk by stress and psychological strain due to for example overwork in everyday life: “I think I have been permanently overstrained all my life.” (CHDP02), concern for relatives and family: „I have a ten year old daughter. What will happen to her?” (ADP02) or disease burden: “Well, there are two areas that need to be worked on. There is my depression, and these signs of psychosis.” (PSYP01).

Strategies described by respondents regarding the management of disease risk relate to actively influencing one’s own health and maintaining quality of life by leading a health-promoting lifestyle: “I do my best to live a healthy lifestyle, eat healthy food, do sports.“ (CHDP03) and by continuing everyday life and one’s social role: “Continuing everyday routines, that’s what is important.” (ADP05). Following on from the central explanatory model of stress as the cause of an existing disease risk, stress avoidance or stress reduction are central strategies, universal to respondents in all clinical areas addressed in this study.

The orientation of strategies is decisively influenced by the therapeutic interventions and preventive options available with regard to the prevention of the onset of a disease or the positive influence on the course of a disease. For three of four indications included in this study (CHD, FBOC, and PSY), these strategies range from surgical interventions and drug therapies to psychotherapeutic and educational approaches. For AD, to date, no effective prevention or cure exists, even at an early stage of risk prediction. However, knowing about the risk can offer the opportunity to make provisions in terms of organisational and existential matters: “Everything is prepared. […] If I got Alzheimer’s dementia tomorrow, I would have everything organised.” (ADP03).

##### Role of Health Care Professionals

The handling of information about one’s own disease risk and the development of strategies for action are thus decisively related to biographical and personality-related relevance systems, information needs and explanatory models.

Do the data also provide insights into the role of consulting physicians in connection with risk perception, processing and risk-adjusted decision-making? The respondents’ comments on the consultation on risk prediction and possible preventive treatment they had experienced ranged from statements of complete satisfaction and feeling well-informed, to the condition of feeling as clueless as before the consultation: “I feel totally well advised.” (FBOC08) or “I know just as much as I did before.” (ADP08).

Patients critically reflect on the counselling situation and the information content, and compare it with their own needs. The role assignment to health care professionals in dealing with a health risk is also negotiated by patients: “I kept asking what I could do about it but she never really gave me any answers.” (ADP08)

A central expectation towards health care professionals in this context is their empathy and understanding with regard to the individual situation of those seeking advice: “A doctor, even if he can’t help, a doctor should be a person who is able to listen.” (FBOCP05). There is also a desire for communication “at eye level”. The power-relation in patient-doctor communication is addressed in various contexts and forms an important category, as it can adversely affect the use of consultation or participation in decision-making processes.

“But on the other hand, it’s actually very important, well it happened now already, um, twice, that something important just showed up in the results .., about which my doctor didn’t talk to me.”

(ADP05)

“I just don’t trust doctors anymore. Oh God, I have experienced so much that I prefer using my own head. […] Things you experience are not always that enjoyable, you know?”

(FBOCP01)

With reference to power relations and participatory decision-making in risk diagnostic counselling and preventive treatment, people seeking advice describe how they feel restricted in their freedom to act. For example, they felt that their choices were influenced by strategic rhetoric they experienced in conversations with their doctors: “It would be better if there were people who helped you in your interest, without giving you the feeling of pushing you towards something they want.” (FBOCP01). These findings emphasise the relevance of asymmetric power structures between patients and health care professionals, due to an imbalance in medical knowledge and expertise, for SDM in the course of medical consultations.

Positive experiences with medical consultations in the predictive field, however, can enable relationships of trust with medical services in general, and with practitioners in particular:

“A trusted relationship with my doctor is essential to me. Now I ended up with a doctor I don’t have any connection to. And in that case… well, with her, I would rather not talk about sensitive stuff.”

(CHDP03)

“Where it actually kicked in for me were my therapy sessions. […] Because of them, I was able to see things more clearly.”

(PSYP10)

These results can contribute to the expansion of the concept of HL by including essential aspects that are relevant for the promotion of HL, especially in the setting of risk prediction. The data emphasise the self-reflected way of persons at risk in dealing with risk information, their own biography and identity in the context of risk, personal definitional concepts of risk and illness, and their perception of risk diagnostic consultations. These results serve as starting points to enable health literate decision-making and action-taking in medical risk context. The reflection of key situations in recognising risk, processes of understanding risk and the negotiation of strategies in dealing with risk are aspects that deserve consideration with respect to identity formation, and with regard to communication strategies in the context of SDM in medical consultations and beyond. Practical implications, both for the expansion of the concept of HL and for early diagnostic consultation practice, will be discussed in the following.

## 4. Discussion

The data of our study provide unique insight into the tension fields between HL, risk, and predictive medicine by adopting the perspective of our interviewees – the so-called “persons at risk”. In this way, our findings can make an important contribution to research on HL, and hereby enrich its theoretical anchoring.

Our findings raise exciting questions about (1) the definition of HL from a bottom-up perspective, (2) the co-construction of HL within the communication process, paying particular attention to the effects of strategic/persuasive communication on SDM, and (3) HL instruments that may have a positive impact on both health system and lifeworld in the context of risk. In the following, we will illuminate each of these fields, grounded in both theoretical and empirical considerations.

### 4.1. Defining HL from a Bottom-Up Perspective: Jumping the Frame of Dealing with Health Information and Opening up a Space for a More Holistic Approach

Our findings support the definition of HL by WHO [2,3] as a complex human competence, which is impacted by different factors. Especially in the context of risk, HL can be described, from the perspective of respective individuals, as a way of balancing between different sources of risk information: the physician or the health system, the Internet and other media, somatic feelings, explanatory models, biographic experiences, and everyday life (in terms of subjective quality of life) which evolve in the context of their individual lifeworlds.

**Figure 2:**
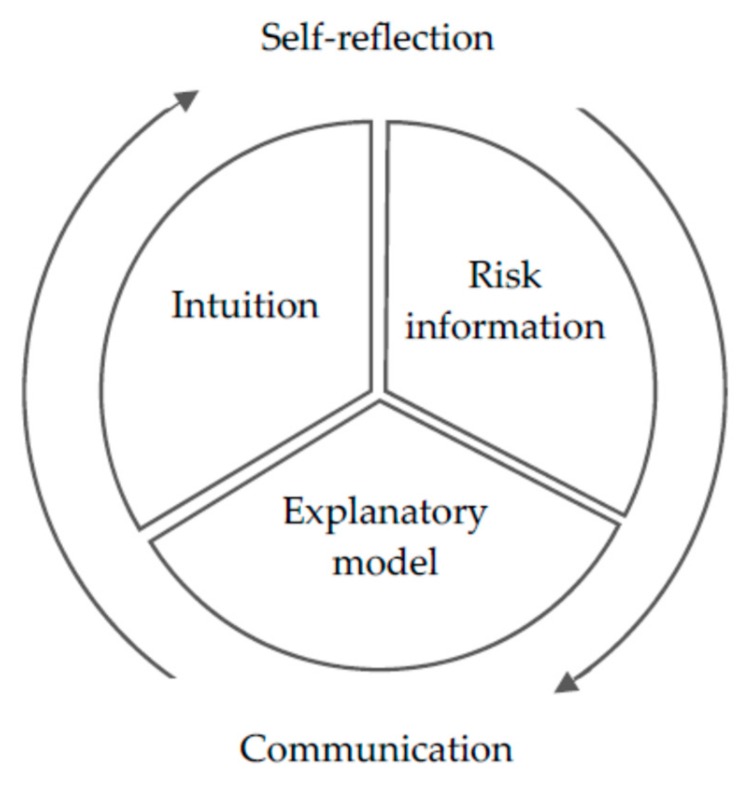
Model of HL in the context of risk– co-construction of risk interpretation and meaning.

In the preparation phase of our research, we used the integrated model by Sørensen et al. [2] as a template and an orientation framework for the development of our study design and the interview-guide, assuming that HL in the context of risk would follow the same or a similar logic. Nevertheless, after first data emerged and was analysed, we quickly realised that an interdisciplinary and multidimensional theoretical embedding will be needed in order to grasp the great amount of symbols and meanings generated throughout the research process. We needed to situate our findings within the field of the HL research (individual and organisational, critical and relational) but also in the sphere of health communication and SDM as well as social science research. The interplay between risk information, the individual explanatory model of risk, intuition, and the ability to reflect on all three aspects (Figure 2) clearly illustrates the necessity to merge different theoretical approaches. This model depicts the understanding of HL from a bottom-up perspective.

On the one hand, this model emphasises the importance of learning more about the patients’ explanatory models of sickness [41] and risk as a way to grasp relevant information in the context of their lifeworld. On the other hand, it refers to Slovic’s [39] theory on risk mentioned above – risk probabilities as part of the analytical system and intuition as part of the emotional system which both on an equal stance enable individuals to make decisions about risk. Furthermore, the dimension of reflection highlights the importance of different domains of critical HL, as described by Chinn [17]: critical appraisal of information, understanding social determinants of health and collective action. These findings are not pioneering in health research; however, they shed a different light on the field of HL in the context of risk perfectly illustrating the “mismatch between ‘biomedical’ and ‘lifeworld’ agenda” [42].

Based on these findings and building on already existing work [7,43,44], we propose a complementary definitional perspective to the concept of HL, employing an ethnographic and health sciences bottom-up approach.

### 4.2. HL as Communicative Action?

In the process of data analysis, the interaction between lifeworld and system turned out to be central for understanding HL in the context of risk from the interviewees’ perspective. For a sound theoretical anchoring of this finding, the philosophical foundations of the theory of communicative action by Jürgen Habermas [45] were considered an appropriate and fruitful background for the theoretical embedding of the empirical data, seeking to bridge action and systems theories. Furthermore, this approach incorporates the notion of power in health communication, which emerged as a central issue when discussing the role of critical HL in the context of predictive and preventive medicine. According to Habermas, actors’ coordination of actions based on common norms is not self-evident, but must always first be reached by mutual agreement between the parties involved; the way in which this happens is through linguistic communication.

In the context of HL and risk communication, the existing literature provides insight into the common norms underlying communication or SDM in medical contexts. But what do we know about the way the actors (professionals and patients) coordinate and negotiate their health-related actions?

Categories like self-reflection, agency, interactions (in the context of patients’ lives and health system) can be fruitfully used to draw an analogy to the Habermasian tensions between (a) lifeworld and system and (b) communicative and strategic action, and provide an inspiring theoretical framework to contextualise the risk-encounter in terms of HL. In the following, we will relate our findings to the theory of communicative action, arguing that this allows for an additional, practice-related and intervention-oriented approach in operationalising and doing research on HL.

#### 4.2.1. Lifeworld and System

The interviewees’ risk narratives play a central role in our empirical findings. On the one hand, we see the personal risk narrative which reveals the social integration of the new status as ‘person at risk’ within one’s own lifeworld. On the other hand, we hear the individuals’ interpretations of the professionals’ narratives, which provide an insight into the assimilating mechanisms of the health system, giving the ‘person at risk’ a certain system-relevant role and access to prediction and prevention. Speaking with Habermasian terms, the system is “colonising” the lifeworld labelling a certain statistical probability as risk and attributing to a still healthy person the status of “person at risk”. In this sense, we can see HL as communicative action where validity claims about risk are made and negotiated. Therefore, the ability to integrate the systemic knowledge into the individual lifeworld and vice versa is an integral part of HL as a dynamic process in both individuals (including the professionals) and the system. Based on this we are prone to see HL as a communicative action that enables professionals, patients, and their relatives to use the risk consultation for negotiating the lifeworld and system narratives and achieve a social and systemic integration of the condition of ‘being at risk’.

In line with the Habermasian theory of communicative action, we therefore believe in the necessity to combine the action- and systems theoretical perspective while doing research on health at risk. Especially in the context of HL, such a theoretical bridge is essential in order to integrate both bottom-up and top-down research perspectives, while addressing the concept and allowing for a holistic approach to health risk and communication.

#### 4.2.2. Communicative and Strategic Action

The Habermasian theory differentiates between two types of rationality – the strategic and the communicative reasoning. Communicative action is oriented towards understanding, consensus and balance; strategic action towards manipulation and personal goal achievement. The interview data also mirror this tension; individuals identified situations in which they felt being persuaded to choose for a certain option of risk prediction or prevention. The empirical data show that individuals describe the communicative action as “communication at eye-level”, which harmonises the agendas of both actors – patients and doctors. Individuals also detect and describe in detail consultation situations in which they felt like a victim of strategic action and communication.

In the context of risk communication in predictive medicine, we should address the ethical question of wishful thinking with regard to HL from both patients’ and professionals’ perspectives – should HL perform a communicative or a strategic role? Our data showed that individuals at risk see HL as a process of communicative action in the context of SDM; it will be particularly interesting to learn more about the perspective of professionals in this context – do they see the goal of the communication in uniting both agendas (in the same sense as SDM), or do they (unconsciously) engage in persuasive rhetoric? Greenhalgh et al. found that:

“Lack of trust, intense pressure of time, mismatch of agendas (biomedical versus lifeworld), firm expectations of a specific outcome (e.g., referral, prescription) and profound power imbalances all promote strategic action (i.e., speech that seeks consciously or unconsciously to manipulate an outcome) rather than communicative action (i.e., sincere efforts to achieve understanding, and reach consensus) by all parties.” [42] (p. 1170)

In this sense, we are deeply convinced that the understanding of the concept of critical HL should be expanded with one further aspect or category – the ability to engage in communicative action and to detect and reflect on strategic action in the process of the risk consultation. Communicative action requires symmetry. In the case of health at risk we need to critically review the validity claims of both parties. Carel & Kidd [46] argue that ill persons are particularly vulnerable to *epistemic injustice*, while health professionals are considered to be epistemically privileged, and the structures of the health system encourage this condition of epistemic injustice. We suggest that this concept may be enormously fruitful when discussing HL in the context of risk on both theoretical and practical level, drawing the attention to the ethical dimensions of HL-promotion.

Following this argumentation line, future research needs to ask further questions on the prerequisites for communicative action in the field of predictive medicine, where lack of certainty is omnipresent: Which are the major barriers to HL as communicative action?

### 4.3. Intervention-Oriented Theory on HL as Communicative Action – Draft and First Ideas

Broadening the definition of HL, emphasising the interaction as the space within which HL is manifested and may be promoted offers a new perspective on the development of instruments for measuring and promoting HL. Based on our findings and the theoretical considerations above, we suggest the following impulses for future research and intervention development.

#### 4.3.1. Interaction as a Target

Based on our empirical findings and their theoretical embedding, we recommend moving the focus on the process of interaction between physicians and patients, not only during the risk consultation itself, but also during preparation and follow-up processing. The results of this study suggest that we should rethink the way of designing HL promotion interventions (especially in the context of risk) which usually aim at contributing to the “accurate understanding” of numbers and statistics. Instead, we should turn our attention also to the consultation encounter itself, enabling patients and professionals to engage in communicative action, detect and disclose strategic communication and reflect on both medical and lifeworld-oriented explanatory models of risk and its consequences.

One possible way to take up the patient’s explanatory model on the one hand and encourage him/her to engage in communicative action on the other is to explore the key situation of risk during the consultation process. The nature of the key situation can provide information about the socio-cultural embedding of the individual understanding of risk. The processing of the key situation in the counselling situation can strengthen a person’s health competence and support professionals in conveying risk information in a patient-centred manner. Discussing the key situation and the explanatory model of risk behind it can contribute to bridging the space between ‘patienthood’ and ‘physicianhood’ [47] (p.352).

#### 4.3.2. (Self-)Reflection as a Tool

One of the key results of the current study is the necessity to add a new, additional dimension to the concept of critical HL in the context of risk – the ability to detect and reflect on strategic communication within the risk-consultation. Furthermore, the competence to integrate the status of being at risk into the individual everyday world and identity, translating medical and systems knowledge into one’s lifeworld experience, has turned out to be a central resource for approaching HL from a bottom-up perspective. However, how can both types of competences be promoted in the course of the consultation?

One possible tool is to integrate self-reflection components in the process of providing, receiving and negotiating risk information. In the course of our research, we found that patients’ narratives do not only reveal information on their medical risk status, but also on the way they have understood and translated this information into their life-world language. Moreover, their accounts may also provide evidence on the strategies individuals have adopted in order to handle the risk information and its consequences. We believe that integrating these narratives in the risk consultation may lead to an increased patient sovereignty [48], more effective communicative action, and extended HL.

#### 4.3.3. Individual and Organisational HL at Once

Our results show that individuals perceive, define, and analyse the role of the health system in the process of risk negotiation primarily through the lens of their interactions with professionals in terms of communication and treatment. With regard to SDM, our interviewees’ experiences of strategic communication by healthcare professionals also underscore the importance of paying attention to prevailing asymmetric power relations in healthcare encounters; ‘expert knowledge’ concerning risk was perceived as a sole privilege of the professionals’ role. In line with other authors, this encouraged us to ask for a more holistic approach to HL promotion on both an individual and an organisational level. For example, Samerski describes individual HL as “a bricolage of different forms of knowledge” [7] (p. 4). Greenhalgh et al. argue that a “failure to play both system and lifeworld roles effectively” [42] (p. 1184) may lead to distorted communication. Carel & Kidd [46] propose a ‘phenomenological toolkit’ to support symmetry and epistemic justice in encounters between patients and healthcare professionals, and to reconcile the patient’s experiential first-person narratives with the ‘objective’ third-person accounts characteristic of the medical world. We will support and further develop this argumentation line, claiming that ‘colonising’ individuals’ lifeworlds with medical and system-centred risk information is a too narrow interventional concept for HL promotion and we believe that in the doctor-patient relationship, more space should be reserved for communication, and for the patient’s lifeworld.

## 5. Limitations and Reflection

Throughout different stages of the research process, this study faced some challenges and limitations:

### 5.1. Methods and Setting

Methodologically, the process of theoretical sampling and the definition of the inclusion and exclusion criteria deserve consideration. In this study, only individuals were included who had been attested an “objective” and medical risk (e.g., genetic mutation). Nevertheless, during the analytical process we realised that the perceived “subjective” risk - which is not based on medical factors and statistics, but on lifeworld-knowledge, intuition, and experience – is as important as the “objective” risk. For future research on risk and HL we suggest also including individuals who believe to be “at risk” even if they cannot prove it in terms of medical documentation.

### 5.2. Recruitment and Sample

By interviewing only people who have participated in early medical diagnosis procedures, the focus of this study is narrowed. In consequence, persons not involved in services of early diagnostics for any reason (e.g., because they do not have access or willingly reject making use of them) are excluded. The findings of this study can therefore not be generalised unrestrictedly to persons ‘at risk’. Participants’ sociodemographic data showed that the sample of this study was rather homogeneous in terms of educational and social background. Therefore, future research on this subject should incorporate recruitment strategies that ensure a more diverse sampling structure with regard to sociodemographic background and experiences with pre-clinical diagnostics.

### 5.3. Analysis

This article is based on comparative, interpretative analysis of data from four clinical fields. Contrary to what we expected, we were not able to recruit 10 individuals at risk of developing Coronary Heart Disease, and hence conducted only three interviews. We therefore did not reach thematic saturation [35] in the first analytical step. We were nevertheless able to identify some core themes for this group, relate them to already existing research, and then use the key messages as an orientation framework for interpretative analysis. Our experiences will inform a reflexive, methodological discussion on the criteria of defining individuals “at risk” of developing CHD (currently under preparation).

### 5.4. Research Environment

Apart from these concrete study limitations, we should pay attention to a more general one, which can be seen as both limitation and challenge – the academic/research environment within which qualitative empirical research on health is being conducted, presented and published. In a medically oriented environment, there is a common sense of doing research in a standardised manner with a linear research process, designed to answer pre-defined hypotheses. A circular research process, defining research questions and using the empirical research to create hypotheses and to generate a theory grounded in data, is still not very common. Researchers hence need to plan additional resources for defending, explaining and legitimising their qualitative exploration-oriented approach within the research environment, and in cooperation with the medical team.

## 6. Conclusions

In our study, we used a qualitative, open methodological approach to investigate the role of HL among persons confronted with a potentially increased disease risk. We identified three central categories that shape individual HL: key situations of risk awareness, the understanding of disease risk, and risk-related agency. These categories are interrelated and play an important role in the process of making meaning of one’s risk, coping with it, and integrating it into one’s identity, health-related behaviour, and life plan. There are several implications for clinical practice, theory building, and future research.

In terms of clinical practice and intervention development, our findings are of vital importance with regard to patient information and risk communication. Our interviewees’ narratives showed that the process of risk negotiation is characterised by introspection and self-reflection, and is closely connected to individuals’ interactions with healthcare professionals. Their rich accounts provide a foundation for the development of practical guidance to support HL in the context of risk in clinical patient-doctor interactions. The way risk is communicated and framed will strongly affect a person’s perception of agency in the sense of autonomy and manageability. This includes individual strategies of information management, decision-making, and acting in view of an increased disease risk. These insights emphasise an understanding of HL as a communicative action, and as a co-construction between the individual, the healthcare professional, and the healthcare system. Hence, actively including patients’ narratives in risk counselling encounters (e.g., by exploring key situations of a person’s confrontation with the respective risk) can be conducive to an effort for more power balance and ‘epistemic fairness’, and for supporting HL in the context of risk prediction and prevention.

In terms of the theoretical underpinnings of HL and future research directions, the results of our study can contribute to an expanded concept of HL, including essential aspects of relevance for the context of risk prediction. Our findings provide insight into individual manifestations of being health- and risk-literate beyond medical information or statistical skills. The interviewees’ risk narratives reveal their very individual journey of understanding, evaluating and applying risk information. We therefore believe that our study will be a valuable complement to the research landscape in terms of theory building and conceptual reflection on the meaning of HL. It can enrich existing work with perspectives on HL grounded in people’s narratives and ethnographic data, hereby contributing to the theoretical grounding of the concept. In methodological terms, future studies may benefit from a more extensive consideration of qualitative designs, in particular ethnographic and participatory approaches, in order to allow for a more open, resource-oriented approach to HL. Moreover, the results of our study can serve as a basis for further research on HL as a communicative element between patients or persons in search of advice and medical professionals; and they can offer starting points for communicative action as a means to realise individual and organisational HL.

## Figures and Tables

**Table 1:** Interviews conducted and included in the data analysis.

	FBOC	AD	CHD	PSY	In Total
Planned	10	10	10	10	40
Conducted	10	11	3	10	34
Included	10	10	3	10	33

## Footnotes

1. When using the term bottom-up approach in this article, we refer to the attempt to take the perspective of those being studied (the so-called persons at risk) without imposing specialist-driven definitions of health, risk, and HL (top-down approach). Instead of empirically testing existing pre-defined scientific concepts, we put the emphasis on health, risk and HL as understood and enacted by our interviewees.

2. The systematic review in the field of familial breast and ovarian cancer is under revision and will be published in 2020.

3. The collaborating partners were involved in the different phases of the project as follows: sampling and recruiting (clinical staff and the authors), data collection in terms of conducting interviews and taking field notes (LH, ML and SJ), data analysis for each clinical field (LH, ML and SJ), and interpretative analysis across the four clinical fields (LH, ML and SJ).

4. Before the interview, all potential study participants were provided with information concerning the aims, methods (including details on the interview procedure, possible harms and benefits, and the continuing opportunity to withdraw from study participation), the exploitation of the research results, and data protection. At the outset of the interview, all participants signed a written consent form.

5. More detailed information on strategies to minimise potential distress for participants, including a reflection on methodological and ethical issues in qualitative research on health risks, can be found in [28].

6. An appropriate description of the body-maps would be beyond the scope of this article. To avoid an oversimplified presentation, we will therefore refrain from providing examples. The in-depth analysis of the body-maps and their role in the process of theory generation will be thematised in a separate article.

7. ‘Saturation’ can be defined from different perspectives and on diverse levels of research [35,36]; in line with our methodological approach, we refer to the model of ‘inductive thematic saturation’ [35] which relates to the emergence of new codes or themes during data analysis.

## Appendix A

**Table A1:** Institutions involved in the recruitment process.

**Familial Breast and Ovarian Cancer:** Zentrum Familiärer Brust- und Eierstockkrebs, **Rita Schmutzler und Kerstin Riehm**	**Alzheimer’s Disease:** Zentrum für Neurologie und Psychiatrie, Klinik für Psychiatrie und Psychotherapie, **Frank Jessen und Ayda Rostamzadeh**
**Coronary Heart Disease:** Schwerpunkt Allgemeinmedizin, **August-Wilhelm Bödecker und Jörg Robertz**	**Psychosis:** Früherkennungs-und Therapiezentrum für psychische Krisen, Klinik und Poliklinik für Psychiatrie und Psychotherapie, **Theresa Haidl und Mauro Seves**

## Appendix B

**Table A2:** Inclusion and exclusion criteria in four clinical fields.

Inclusion Criteria	Exclusion Criteria
**Coronary Heart Disease**	
Adults (a) with an increased risk of developing coronary heart disease (CHD) or (b) those suffering from clinically manifest CHD (according to the cardiovascular disease risk charts of the European Society of Cardiology [49]).	- Age < 35 years - Severe physical disease (except CHD and diabetes mellitus) - Mental illness (e.g., dementia, substance dependence, psychosis)
(a) adults without known CHD	
Age: women > 60 years; men > 50 years	
Hypertension (blood pressure > 140/95 mmHg) and/or total cholesterol > 200 mg/dl and/or smoking and/or diabetes mellitus	
(b) adults with known CHD	
Women and men with known KHK, smoking and/or blood pressure > 140/95 mmHg and/or LDL cholesterol > 100 mg/dl and/or diabetes with HbA1c > 7.5%.	
Written declaration of consent	
German language skills that allow the interview conduction	
**Psychosis**	
- Adults who fulfill the clinical high-risk criteria for psychosis (basic symptom criteria (SPI-A) and/or the ultra-high risk criteria (SIPS))	- Age < 18 years - Increased risk based only on instruments of self-assessment - Known presence of a traumatic event - Current clinically relevant depressive episode, anxiety symptoms or suicidal tendencies
- Written declaration of consent	
- German language skills that allow the interview conduction	
**Alzheimer’s Disease**	
- Written declaration of consent German language skills that allow the interview conduction	- Dementia Indications of a non-AD neurodegenerative disease such as: Parkinson’s disease, Lewy’s body dementia, frontotemporal lobar degeneration, very rapid cognitive deterioration within a few weeks or months (classically indicative of a prion disease, neoplasia or metabolic disorder) or brain tumour
**Clinical Criteria for the Diagnosis of an MCI (According to NIA-AA Criteria):** Cognitive impairment (self or foreign medical history reported) Objective impairment in one or more cognitive domains Maintain daily life activities (ATLs) No dementia	
**Clinical Criteria for the Diagnosis of an SCD (According to the Criteria of Jessen et al. 2014):** Subjective and persistent (not acute) deterioration of cognitive performance compared to the original starting level Neuropsychological test battery, which is used for MCI or prodromal AD, shows a positive response within the age range, gender- and education-adjusted norm group lying findings	- Current clinically relevant depressive episode (GDS >11), other serious psychiatric disorders or suicidal tendencies - MCI, prodromal AD or dementia - impairments caused by a psychiatric* or neurological disease (excluding AD), somatic disease, medication or substance abuse can be explained * mild subsyndromal depressive symptoms or anxiety symptoms are not considered an exclusion criterion
**Familial Breast and Ovarian Cancer**	
- Group 1: Carrier of a BRCA1 or BRCA2 mutation	- Age < 18 years mild cognitive disorders or Alzheimer’s dementia - Current clinically relevant depressive episode, anxiety symptoms or suicidal tendencies
- Group 2: Carrier of a mutation in a moderate risk gene (e.g., CHEK2)	
- Group 3: No mutation detection in one of the known risk genes, but increased mathematical risk of disease due to own and family anamnesis	
- Written declaration of consent	
- German language skills that allow the interview conduction	

## Appendix C

**Table A3:** Sociodemographic sample description per clinical field.

		FBOC (n = 10)	PSY (n = 10)	AD (n = 10)	CHD (n = 3)	TOTAL (n = 33)
**Gender**	Female	10	4	3	1	18
	Male	-	6	7	2	15
	Other	-	-	-	-	
**Age**	18–30	1	9	-	-	10
	31–40	5	1	-	-	6
	41–50	3	-	-	1	4
	51–60	-	-	1	1	2
	61–70	-	-	8	1	9
	≥ 71	-	-	1	-	1
**Marital Status**	Not specified	-	-	1	1	2
	Single	3	9	1	2	15
	Married	6	1	5	-	12
	Widowed	-	-	1	-	1
	Divorced	1	-	2	-	3
	Separated	-	-	-	-	-
**Living Conditions**	Alone	1	2	2	1	6
	Shared apartment	-	4	-	-	4
	With partner	5	1	5	2	13
	With relative	1	2	2	-	5
	With partner and relative	3	-	1	-	4
	Other	-	-	-	-	-
**Cultural Background**	German	7	7	9	3	26
	Bi-cultural	2	3	1	-	6
	Other	1	-	-	-	1
**Mother Tongue**	German	7	8	9	3	27
	Bi-lingual	2	2	1	-	5
	Other	1	-	-	-	1
**Education**	Abitur ^1^	8	8	4	2	22
	Fachhochschulreife ^2^	-	-	3	1	4
	Mittlere Reife ^3^	2	2	1	-	5
	Polytechnische Oberschule ^4^	-	-	-	-	-
	Haupt-/Volksschulabschluss^5^	-	-	2	-	2
	No school certificate	-	-	-	-	-
	Other	-	-	-	-	-
**Employment Status**	Full-time	5	6	1	-	12
	Part-time	4	-	2	2	8
	In training/study	-	3	-	-	3
	Homemaking	-	-	-	-	-
	Retirement	-	-	5	-	5
	Jobseeker	-	-	1	-	1
	Unemployed	-	-	1	1	2
	Work disability	1	1	-	-	2

FBOC—Familial breast and ovarian cancer. PSY—Psychosis. AD—Alzheimer’s disease. CHD—Coronary heart disease. ^1^ Abitur = Highest degree of German school system, general or subject-specific higher education entrance qualification. ^2^ Fachhochschulreife = Degree of German school system qualifying for general or subject-specific upper secondary school entrance. ^3^ Mittlere Reife = Middle degree of German school system qualifying for vocational school or comparable. ^4^ Polytechnische Oberschule = School form of former German Democratic Republic, comparable to degree of “Mittlere Reife” ^5^ Haupt-/Volksschulabschluss = Qualification of a general school form of middle education.

**Table A4:** Sociodemographic sample description per interview partner.

Clinical Field	Sex	Age	Marital Status	Living Conditions	Cultural Background	Mother Tongue	Religious?	Highest School Leaving Certificate	Branch/Profession-al Activity/Education	Employment Relationship	Long-Term Med. Treatment	Chronic Disease
FBOCP01	Female	41 to 50	Divorced	With rel.	German	German	No	Abitur ^1^	Social area	Fulltime	Yes	No
FBOCP02	Female	31 to 40	Unmarried	With partner	German	German	Yes	MR ^3^	Economics	Part-time	Yes	Yes
FBOCP03	Female	31 to 40	Married	Partner & rel.	Bi-cultural	G. & others	Yes	Abitur ^1^	Social area	Part-time	No	No
FBOCP04	Female	18 to 30	Unmarried	With partner	German	German	No	Abitur ^1^	Art & culture	Disabled	No	No
FBOCP05	Female	41 to 50	Married	Alone	Bi-cultural	G. & others	n.a.	Abitur ^1^	IT	Fulltime	Yes	Yes
FBOCP06	Female	31 to 40	Married	With partner	German	German	Yes	MR ^3^	Health area	Fulltime	Yes	No
FBOCP07	Female	41 to 50	Married	With partner	German	German	Yes	Abitur ^1^	Other	Fulltime	Yes	Yes
FBOCP08	Female	51 to 60	Married	Partner & rel.	German	German	Yes	Abitur ^1^	Health area	Part-time	Yes	Yes
FBOCP09	Female	18 to 30	Unmarried	With partner	Other	Other	Yes	Abitur ^1^	Health area	Part-time	No	No
FBOCP10	Female	31 to 40	Married	Partner & rel.	German	German	Yes	Abitur ^1^	Health area	Fulltime	Yes	No
ADP01	Female	61 to 70	Divorced	Alone	German	German	Yes	FH-Reife ^2^	Health area	In pension	Yes	Yes
ADP02	Female	61 to 70	Married	With partner	German	German	No	Abitur ^1^	Social area	In pension	Yes	Yes
ADP03	Male	61 to 70	Married	Alone	Other	Other	No	Abitur ^1^	Other	In pension	Yes	Yes
ADP04	Male	61 to 70	Married	Partner & rel.	German	German	Yes	FH-Reife ^2^	Health area	Fulltime	Yes	Yes
ADP05	Female	61 to 70	n.a.	With partner	German	German	No	MR	Admin.	Part-time	Yes	Yes
ADP06	Male	61 to 70	Married	With partner	German	German	Yes	Abitur ^1^	Science	In pension	No	Yes
ADP07	Female	61 to 70	Widowed	With partner	German	German	Yes	HS ^5^	Other	Unemployed	No	No
ADP08	Female	61 to 70	Married	With partner	German	German	No	Abitur ^1^	Admin.	Seeking work	Yes	Yes
ADP09	Female	51 to 60	Single	With rel.	German	German	Yes	FH-Reife ^2^	Social area	Part-time	Yes	No
ADP10	Female	71 or older	Divorced	With rel.tives	German	German	Yes	HS ^5^	Health area	In pension	No	No
PSYP01	Male	18 to 30	Unmarried	Alone	Bi-cultural	G. & others	No	Abitur ^1^	IT	In training	Yes	Yes
PSYP02	Female	31 to 40	Married	Partner & rel.	German	German	No	Abitur ^1^	Social area	Fulltime	No	No
PSYP03	Female	18 to 30	Unmarried	With rel.	German	German	No	Abitur ^1^	Social area	In training	Yes	No
PSYP04	Female	18 to 30	Unmarried	Shared app.	German	German	No	Abitur ^1^	Social area	Fulltime	No	No
PSYP05	Male	18 to 30	Unmarried	With partner	German	German	No	Abitur ^1^	Social area	Fulltime	Yes	Yes
PSYP06	Female	18 to 30	Unmarried	Shared app.	German	German	Yes	MR ^3^	Other	Disabled	Yes	Yes
PSYP07	Male	18 to 30	Unmarried Unmarried	With rel.	German	German	Yes	Abitur ^1^	Other	Fulltime	Yes	No
PSYP08	Male	18 to 30	Unmarried	Shared app.	German	German	No	Abitur ^1^	Social area	In training	No	Yes
PSYP09	Male	18 to 30	Unmarried	Shared app.	Bi-cultural	G. & others	Yes	Abitur ^1^	Art & culture	Fulltime	Yes	Yes
PSYP10	Male	18 to 30		Alone	Bi-cultural	German	No	MR ^3^	Social area	Fulltime	No	No
CHDP01	Male	51 to 60	Unmarried	Alone	German	German	n.a.	Abitur ^1^	Trade	Unemployed	Yes	Yes
CHDP02	Female	61 to 70	n.a.	With partner	German	German	Yes	FH-Reife ^2^	Economics	Part-time	Yes	Yes
CHDP03	Male	41 to 50	Unmarried	With partner	German	German	Yes	Abitur ^1^	Other	Part-time	No	No

FBOC—Familial breast and ovarian cancer. PSY—Psychosis. AD—Alzheimer’s disease. CHD—Coronary heart disease. ^1^ Abitur = Highest degree of German school system, general or subject-specific higher education entrance qualification. ^2^ Fachhochschulreife = Degree of German school system qualifying for general or subject-specific upper secondary school entrance. ^3^ Mittlere Reife = Middle degree of German school system qualifying for vocational school or comparable. ^4^ Polytechnische Oberschule = School form of former German Democratic Republic, comparable to degree of “Mittlere Reife” ^5^ Haupt-/Volksschulabschluss = Qualification of a general school form of middle education.

## Appendix D

**Table A5:** Data material used for analysis.

	Audio Material	Transcripts	Questionnaires	Body Maps
**FBOC**	10:00 h	351 pages	10	10
**AD**	09:20 h	325 pages	10	8
**CHD**	04:10 h	60 pages	3	3
**PSY**	10:20 h	300 pages	10	10
**Total**	**_~_34:00 h**	**1036 pages**	**33**	**31**

## References

[1] Kickbusch I. (2007). Health Governance: The Health Society. Health and Modernity.

[2] Sørensen K., van den Broucke S., Fullam J., Doyle G., Pelikan J., Slonska Z., Brand H. (2012). Health Literacy and Public Health: A Systematic Review and Integration of Definitions and Models. BMC Public Health.

[3] Kickbusch I., Pelikan J.M., Apfel F., Tsouros A.D. Health Literacy. The solid facts. WHO Regional Office for Europe 2013. https://apps.who.int/iris/bitstream/handle/10665/128703/e96854.pdf.

[4] Oliveira G.S.d., Errea M., Bialek J., Kendall M.C., McCarthy R.J. (2018). The impact of health literacy on shared decision making before elective surgery. A propensity matched case control analysis. BMC Health Serv. Res..

[5] Muhanga M.I., Malungo J.R.S. (2017). The what, why and how of health literacy: A systematic review of literature. Int. J. Health.

[6] Abel T., Sommerhalder K. (2015). Gesundheitskompetenz/Health Literacy. Das Konzept und seine Operationalisierung. Bundesgesundheitsblatt Gesundh. Gesundh..

[7] Samerski S. (2019). Health literacy as a social practice. Social and empirical dimensions of knowledge on health and healthcare. Soc. Sci. Med..

[8] Smith S.K., Dixon A., Trevena L., Nutbeam D., McCaffery K.J. (2009). Exploring patient involvement in healthcare decision making across different education and functional health literacy groups. Soc. Sci. Med..

[9] Altin S.V., Stock S. (2016). The impact of health literacy, patient-centered communication and shared decision-making on patients’ satisfaction with care received in German primary care practices. BMC Health Serv. Res..

[10] Joseph-Williams N., Williams D., Wood F., Lloyd A., Brain K., Thomas N., Prichard A., Goodland A., McGarrigle H., Sweetland H. (2019). A descriptive model of shared decision making derived from routine implementation in clinical practice (‘Implement-SDM’): Qualitative study. Patient Educ. Couns..

[11] Shen H.-N., Lin C.-C., Hoffmann T., Tsai C.-Y., Hou W.-H., Kuo K.N. (2019). The relationship between health literacy and perceived shared decision making in patients with breast cancer. Patient Educ. Couns..

[12] Hauser K., Koerfer A., Kuhr K., Albus C., Herzig S., Matthes J. (2015). Outcome-Relevant Effects of Shared Decision Making. Dtsch. Ärzteblatt Int..

[13] Stiggelbout A.M., Pieterse A.H., de Haes J.C.J.M. (2015). Shared decision making. Concepts, evidence, and practice. Patient Educ. Couns..

[14] Koch K. (2003). Informationen über Krebsfrüherkennung. Was wollen die Patienten?. Med. Klin..

[15] Ishikawa H., Kiuchi T. (2010). Health literacy and health communication. Biopsychosoc. Med..

[16] Schaefer C., Bitzer E.M., Dierks M.L. für den Vorstandes DNGK. Mehr Organisationale Gesundheitskompetenz in die Gesundheitsversorgung bringen! Ein Positionspapier des DNGK. Köln, 15.11.2019. https://dngk.de/gesundheitskompetenz/or-ganisationale-gesundheitskompetenz-positionspapier-2019/.

[17] Chinn D. (2011). Critical health literacy: A review and critical analysis. Soc. Sci. Med..

[18] Nutbeam D. (2000). Health literacy as a public health goal: A challenge for contemporary health education and communication strategies into the 21st century. Health Promot. Int..

[19] Sørensen K., Pelikan J.M., Röthlin F., Ganahl K., Slonska Z., Doyle G., Fullam J., Kondilis B., Agrafiotis D., Uiters E. (2015). Health literacy in Europe: Comparative results of the European health literacy survey (HLS-EU). Eur. J. Public Health.

[20] Alper J. (2018). A Proposed Framework for Integration of Quality Performance Measures for Health Literacy, Cultural Competence, and Language Access Services: Proceedings of a Workshop. Roundtable on Health Literacy; Board on Population Health and Public Health Practice; Health and Medicine Division.

[21] Haidl T.K., Seves M., Eggers S., Rostamzadeh A., Genske A., Jünger S., Woopen C., Jessen F., Ruhrmann S., Vogeley K. (2019). Health literacy in clinical high-risk individuals for psychosis: A systematic mixed-methods review. Early Interv. Psychiatry.

[22] Rostamzadeh A., Stapels J., Genske A., Haidl T., Jünger S., Seves M., Woopen C., Jessen F. (2019). Health Literacy in Individuals at Risk for Alzheimer’s Dementia: A Systematic Review. J. Prev. Alzheimer’s Dis..

[23] Hellstern M., Peltzer S., Genske A., Jünger S., Woopen C., Albus C. (2020). Health literacy in persons at risk of and patients with coronary heart disease: A systematic review. Soc. Sci. Med..

[24] Patton M. (1990). Qualitative Evaluation and Research Methods.

[25] Guest G., Bunce A., Johnson L. (2016). How Many Interviews Are Enough?. Field Methods.

[26] Schütze F. (1983). Biographieforschung und narratives Interview. Neue Prax..

[27] Nohl A.-M. (2017). Interview und Dokumentarische Methode. Anleitungen für die Forschungspraxis.

[28] Lorke M., Schwegler C., Jünger S., Willig M.C. Re-claiming the power of definition—The value of reflexivity in research on mental health at risk. Qualitative Research Methods in Mental Health: Innovative and Collaborative Approaches Borcsa.

[29] (2019). VERBI Software. MAXQDA 2018.

[30] Gastaldo D., Rivas-Quarneti N., Magalhaes L. (2018). Body-Map Storytelling as a Health Research Methodology. Blurred Lines Creating Clear Pictures. FQS.

[31] Merleau-Ponty M. (1966). Phänomenologie der Wahrnehmung.

[32] Stamer M., Güthlin C., Holmberg C., Karbach U., Patzelt C., Meyer T. (2015). Qualitative Studien in der Versorgungsforschung - Diskussionspapier, Teil 3. Qualität qualitativer Studien. Gesundheitswesen.

[33] Lincoln Y.S., Guba E. (1985). Naturalistic Inquiry.

[34] Breuer F., Muckel P., Dieris B. (2019). Reflexive Grounded Theory. Eine Einführung für die Forschungspraxis.

[35] Saunders B., Sim J., Kingstone T., Baker S., Waterfield J., Bartlam B., Burroughs H., Jinks C. (2018). Saturation in qualitative research: Exploring its conceptualization and operationalization. Q. Quant.

[36] Strübing J., Hirschauer S., Ayaß R., Krähnke U., Scheffer T. (2018). Gütekriterien qualitativer Sozialforschung. Ein Diskuss. Z. Für Soziologie.

[37] Denzin N. (1970). The Research Act in Sociology.

[38] Park C.L., Folkman S. (1997). Meaning in the Context of Stress and Coping. Rev. Gen. Psychol..

[39] Slovic P., Finuncane M.L., Peters E., MacGregor D.G. (2004). Risk as Analysis and Risk as Feelings: Some Thoughts about Affect, Reason, Risk and Rationality. Risk Anal..

[40] Nutbeam D. (2008). The evolving concept of health literacy. Soc. Sci. Med..

[41] Kleinman A. (1988). The Illness Narratives: Suffering, Healing, and the Human Condition.

[42] Greenhalgh T., Robb N., Scambler G. (2006). Communicative and strategic action in interpreted consultations in primary health care: A Habermasian perspective. Soc. Sci. Med..

[43] Papen U. (2012). Informal, incidental and ad hoc: The information-seeking and learning strategies of health care patients. Lang. Educ..

[44] Fairbrother H., Curtis P., Doyder E. (2016). Making health information meaningful: Children’s health literacy practices. Ssm Popul. Health.

[45] Habermas J. (1981). Theorie des Kommunikativen Handelns.

[46] Carel H., Kidd I.J. (2014). Epistemic injustice in healthcare: A philosophical analysis. Med. Health Care Philos..

[47] DasGupta S., Charon R. (2004). Personal Illness Narratives: Using Refelctive Writing to Teach Empathy. Acad. Med..

[48] Dierks M.-L., Koppelin F., Müller R., Keil A., Hauffe U. (2001). Brustkrebs-Früherkennung: Einstellungen und Motive von Frauen zur Mammographie. Die Kontroverse um die Brustkrebs-Früherkennung.

[49] Piepolii M.F., Hoes A.W., Agewall S., Albus C., Brotons C., Catapano A.L., Cooney M.-T., Corrà U., Cosyns B., Deaton C. (2016). 2016 European Guidelines on cardiovascular disease prevention in clinical practice: The Sixth Joint Task Force of the European Society of Cardiology and Other Societies on Cardiovascular Disease Prevention in Clinical Practice (constituted by representatives of 10 societies and by invited experts). Eur. Heart J..

